# Ethnoprimatology of the Shipibo of the upper Ucayali River, Perú

**DOI:** 10.1186/s13002-023-00616-1

**Published:** 2023-10-19

**Authors:** Evelyn Anca, Sam Shanee, Magdalena S. Svensson

**Affiliations:** 1 Faculty of Humanities and Social Sciences, Oxford Brookes University, Oxford, UK; 2 Neotropical Primate Conservation, Cornwall, UK; 3 Asociación Neotropical Primate Conservation Perú, Moyobamba, Perú

**Keywords:** Ethnoprimatology, Human–primate interactions, Primates, Indigenous people, Shipibo, Peruvian Amazon, Traditional knowledge

## Abstract

In the Anthropocene, primate conservation can only take place when considering human culture, perspectives, and needs. Such approaches are increasingly important under the growing impact of anthropogenic activities and increasing number of threatened primates. The Amazon rainforest, rich in cultural and biological diversity, where indigenous people play a crucial role in primate conservation, provides ample opportunity to study human–primate interactions and the sociocultural context in which they occur. Human activities threaten the Amazon’s fragile ecosystems and its primates, which play a key role in its maintenance and regeneration. This study focuses on one of the largest indigenous groups in the Peruvian Amazon: the Shipibo. Interviews and participant observation were used to investigate local perceptions of animal presence and depletion, food preferences, and how primates are incorporated into daily life and culture. Since time immemorial and still today, primates remain important in Shipibo culture, mythology, and subsistence. Local Shipibo participants consistently identified the presence of 13 species of primate. Primates were among the preferred species for consumption, pet keeping, and held a fundamental role in mythology, traditional knowledge, and storytelling. Large-bodied primates were often mentioned as being locally extinct, with reports and observations suggesting increasing consumption of smaller-bodied primates. Commonly perceived reasons for primate depletion include noise disturbance, hunting, and population growth, often in parallel. This study sheds light on the cultural context of an area rich in biodiversity, where primates, essential for ecological balance and integral to Shipibo lives and identity, are being depleted. We highlight the need for an inclusive ethnoprimatological approach to conserving primates and preserving indigenous heritage while improving local livelihoods.

## Background

The current human impact on nature is so unprecedented that it has been used to propose a geological epoch of its own, the Anthropocene, defined by the impact of human activities on nature, wildlife and Earth as a whole [1]. As conservationists, we should strive for a better understanding of human behaviours, and the complex motivations behind them, which lead to biodiversity loss, incorporating them into conservation strategies [2]. Among the many taxa threatened by human activities, non-human primates (hereafter primates), our closest living relatives, are severely affected, with ~ 60% of species threatened and > 90% of species in population decline [3, 4]. Humans and primates have interacted for hundreds of thousands of years and these interactions are still seen today in various relationships that occur in shared spaces [5]. Human population expansion, deforestation, habitat destruction, and increasing demand for resources often generates conflict with primates [3, 6]. The field of ethnoprimatology combines biological, social, and cultural aspects of primatology and anthropology [5, 7]. This makes it a rounded approach to study human–primate interactions and the relationships that affect their behaviour and ecology [6, 7].

The distributions of 71% of the world’s primate species overlap with indigenous lands [8], where human–primate interactions are often intertwined with a group’s social and economic activities, culture, and traditional practices. These interactions can be characterized by direct uses such as subsistence hunting and pet keeping [9–12], as well as 'conflicts' such as crop foraging and other disturbances in urban areas, farms, and tourist sites [13–15]. Primates are also a large and favoured part of the diet of many indigenous and local people in the tropics [3, 16]. There are also often symbolic representations of primates in traditional folklore, beliefs, taboos, and mythology [9, 17, 18]. The cultural importance of primates, as reflected in indigenous knowledge, mythology, traditional practices and beliefs, provides valuable information about human–primate interactions and may reveal motivations for different attitudes towards primates and primate consumption preferences, resulting from their cultural and religious attributes [6, 13, 17]. Indigenous knowledge is often ignored by conservationists and replaced by ‘Western’ ways of knowing [19, 20]. This exclusion creates knowledge gaps, denies agency, and can lead to negative sentiment towards conservation among local communities, undermining conservation interventions [19–21]. Without including local knowledge and understanding of the culture of those who have shared the same environment as the target species, these interventions can be one-dimensional and incomplete [21–24]. In line with the growing recognition of the value of traditional ecological knowledge (TEK), for both indigenous people and biodiversity, conservation efforts must actively document and include these dynamic knowledge systems of the environment as an essential component for conservation success [22–24]. As lands used by people and primates increasingly overlap through the expansion of non-indigenous people into indigenous lands, rates of deforestation and ecosystem degradation have also increased [8, 25]. This has far-reaching effects on primates, through the direct loss of suitable habitat and by threatening traditional culture and knowledge which are crucial for the preservation of biodiversity [8, 26]. Studying these interactions and the cultural context in which they occur can help tailor effective conservation strategies, while defending indigenous peoples’ lands, needs, and knowledge [8, 12, 13, 19, 20, 23, 25].

The term ethnoprimatology was originally inspired by human–primate interactions and the important role primates have in the lives of indigenous people in the Amazon [27]. Primate species diversity in the Neotropics is the highest in the world and is largely concentrated in the Amazon, which is also home to a growing population of over 30 million people [3, 28]. Forty-eight per cent of the Neotropical primate range overlaps with indigenous peoples’ lands [8]. Primates hold cultural importance in the lives of various Amazonian indigenous groups, from both anthropological and conservation perspectives, with evidence of human–primate interactions in at least 70 groups in the Amazon, through a range of subsistence practices and dietary taboos relating to primates [29, 30]. A review of these interactions referenced 34 indigenous groups in which primates are incorporated into folklore, magic, and religion, among them creation myths, cautionary tales, and beliefs where the theme of continuity and transformation between humans and primates stands out [29]. With 42% of Neotropical primates threatened with extinction [4], and the intense exploitation of the Amazon, it is crucial to understand human–primate interactions and what defines them, in indigenous communities.

As some indigenous populations shift from traditional practices towards integration with ‘Westernized’ lifestyles and technologies, understanding the impact of these changes on biodiversity is vital for primate conservation. Primates are a preferred dietary resource for many indigenous peoples in the Amazon [29–31]. Changes from traditional hunting methods, such as bows and arrows, to shotguns, have resulted in higher extraction rates, in line with growing human populations and associated with increased demand for food and to bolster income [9, 32, 33]. Hunter preference for larger-bodied species often leads to their depletion and local extinction [31, 34, 35]. As larger-bodied species become scarce, hunters often hunt more smaller-bodied species, depleting remaining animal populations [34–39]. In addition to human population growth, integration into economic markets has been linked to increased commercial hunting and has implications for cultural identity [38, 40, 41]. Nonetheless, other indigenous groups may embrace the change, such as the Lacandon Maya, who have reduced traditional primate hunting to concentrate on profit from tourism [42], and a Tikuna community where a hunting ban on woolly monkeys alongside tourism increased local interest in conservation and sustainable forest resource management [10].

Since Cormier’s review [29] on human–primate relationships in the Amazon, when few studies specifically focused on the topic, literature has expanded within the field of ethnoprimatology [30]. Ethnoprimatological studies with indigenous Amazonians have tended to focus on smaller indigenous communities living in less disturbed environments showing the significance of primates in cultural identity, subsistence and uses in social activities [12, 13, 18, 43, 44]. However, understanding the resilience of cultural values and adaptations of traditional practices in larger indigenous populations (both numerically and geographically) [10, 32, 33] living in more disturbed places has important implications for conservation as they become more integrated into wider society and market economies. Ucayali is home to the Shipibo-Conibo, one of the largest and best-known indigenous groups in the Peruvian Amazon. The Shipibo-Conibo are found to the northeast and southeast of Perú’s second largest Amazonian city, Pucallpa. The Shipibo-Conibo population in Perú was over 34,000 people as of 2017 [45], and their language, Shipibo, from the Panoan family of languages, is the sixth most spoken in the country. The name Shipibo-Conibo is derived from the words *shipi* (monkey), *coni* (eel), and *bo*, suffix for plural, i.e. monkey-people and eel-people, respectively [46]. Historically, the Shipibo and Conibo were two separate groups which gradually merged through intermarriage and migration and will be referred to as Shipibo herein as is their own custom and preference. The Shipibo practise shifting agriculture and rely on farming, hunting, and fishing for subsistence and economic needs, with fish being their main protein source [45, 46]. While Shipibo lands are home to a high diversity of primate species, to our knowledge no previous study focusing on the cultural role of primates has been undertaken with this group. Human–primate interactions in Shipibo communities are evidenced through their extractive uses and related taboos [29, 47]. Behrens [47] studied the categorization of food among the Shipibo, where primates were mentioned as a domesticated animal, regularly consumed species, and linked to consumption taboos [47]. Morin’s ethnographic work [46] documented the use of primates as food, pets, and their parts in jewellery as well as myths on the human origin of primates [46]. These findings from over 30 years ago offer a glimpse into the levels of human–primate interactions with indigenous Shipibo and pave the way for further exploration of these relationships and their implications.

In this study, we explore the ethnoprimatology of the Shipibo as part of a holistic approach to learn about human–primate interactions and the structure of primate populations along the upper Ucayali River. Our aim is to better understand and expand the knowledge on the roles and uses of primates in Shipibo culture, diet, mythology and beliefs. Using free listing interviews, we further investigate the presence and depletion of local wildlife, as well as reasons for animal depletion, as perceived and explained by the local Shipibo.

## Methods

### Study site

We carried out our study in the *Comunidad Nativa de Pueblo Nuevo del Caco* (PNC), which is one of five Shipibo communities along the Caco River, a minor tributary of the Ucayali River. The people of PNC and neighbouring native communities are predominantly of Shipibo origin. A small number of migrants from other regions of Perú come to live and work in the area temporarily, although this can last for years. All the communities lie North of the Caco River and share forests and trails that interconnect them, although most travel between villages is made by paddle or motorized canoes (‘peke peke’). The surrounding habitat is highly disturbed old-growth terra firme forest, which has been, and continues to be, subject to selective logging. Forests are interspersed with shifting and fixed agricultural and pastoral lands. The PNC was legally recognized in 1974 as a *Comunidad Nativa* (Native Community) and holds collective land title to an area of 6,985 ha [45, 48]. According to the most recent census conducted by the community in 2021, the community has ~ 120 families [Denis Napo, Pers. Comm. To EA]. As found throughout Shipibo-Conibo communities, the people of PNC self-identify as Shipibo, not distinguishing between Shipibo and Conibo origins.

### Data collection

We collected data between 1 May and 28 June 2022.

**Fig. 1:**
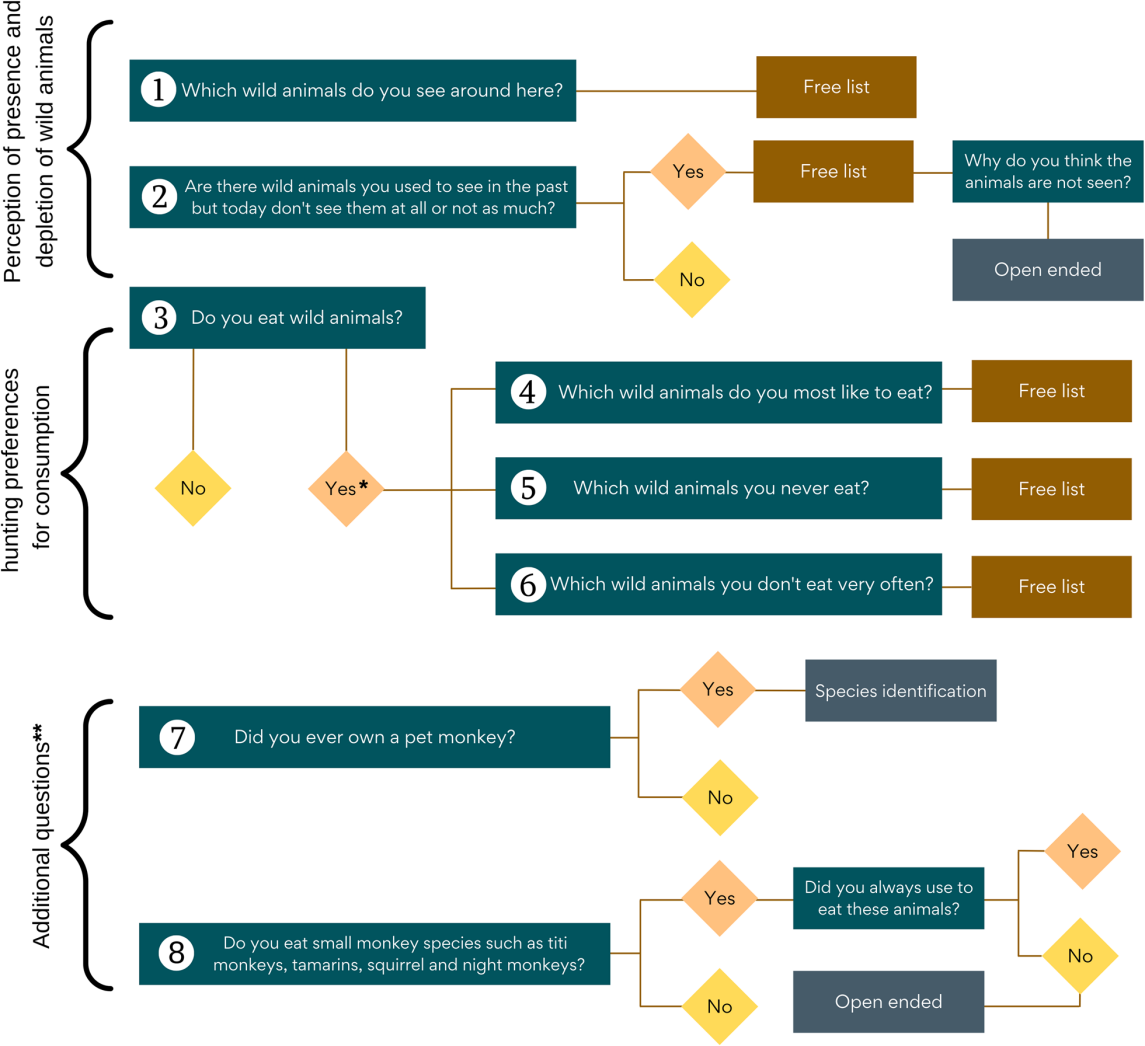
Flow chart showing the free listing and interview questions, translated to English. *100% of the participants responded ‘Yes’. **Question 7 was asked at the end of the free listing interviews; question 8 was a part of the additional survey with the same participants

*Free listing interviews* is a method often used in anthropology and social sciences to capture cultural and cognitive domains, their importance and familiarity [49, 50]. These interviews were used to identify hunting preferences for consumption, and the perception of hunted species’ presence and temporal abundance. We used a multi-species approach (primates and non-primates) in interviews to explore the broader context in which primates are consumed as food and seen in the environment among other hunted animal species. This allowed for comparison with other hunted species, without imposing a scientific taxonomic order [51, 52]. Male and female community members were interviewed. All interviewees were of Shipibo origin, at least 25 years old, and had either grown up in the community or had lived there > 10 years. Prior to interviews, participants gave voluntary oral consent and interviews were carried out at a time and place that suited them, noting they could withdraw at any time. Interviews lasted 10–15 min and were conducted in Spanish. Interviews included two free listing questions and an open-ended question about the perception of current presence and absence of species relative to the past, as well as three free listing questions about consumption preferences (Fig. 1). Yes/no questions were used before the free listing questions to avoid leading participants. Primate species mentioned by participants were confirmed using photo flashcards. At the end of each interview, participants were asked if they owned a primate, and if so, the species was confirmed using the same flashcards (Fig. 1, Question 7). Following observations in the community in the first three weeks, we carried out an additional two-question survey regarding the consumption of smaller primate species (Fig. 1. Question 8).

*Semi-structured interviews* were used to allow more input through open conversations with participants about cultural views, folklore and knowledge of primates, as well as their uses [49, 50]. Semi-structured interviews were conducted separately from the free listing interviews with seven community members aged 38 to 74 (mean age = 61.9). After the free listing interviews, participants were asked if they would agree to participate in a more in-depth interview on primates and their role in Shipibo culture. Most participants responded that they do not know, or do not remember, this knowledge and referred us to elders in the community. As participants in semi-structured interviews were fewer, and selected based on their knowledge, we refer to them as informants. Oral consent was provided prior to each interview, informants were made aware they could withdraw at any time, and notes were taken using a notebook or digital recorder when informants agreed to be recorded. Interviews began with the informant listing all primate species they were aware of around the community, with species ID again confirmed with photographs. Conversations centred on the uses of primates and their derivatives, traditional knowledge and folklore related to primates. The use of primates and their derivatives referred to any primate part, processed or raw (e.g. meat, skin, bones, etc.), as well as prohibitions on such uses. Traditional knowledge was recorded as any information derived from observations, stories and notions about primates, their behaviour and ecology [23, 24]. Under folklore, we focused on myths and traditional beliefs regarding primates; based on Malinowski and Eliade’s perspectives on mythology as cited by Boskovic [53], myths were defined as stories from an ancient past that explain current realities and the creation origins of humans and animals. Traditional beliefs are not explicitly set in an ancient time and result from or justified by myths [53]. In addition, the data were categorized with consideration to what informants referred to as ‘myth’, ‘belief’, and ‘story’.

*Participant observation* through immersion in the daily lives of the community members allowed us to observe the community’s use of primates, and related behaviours that may not have been reported during interviews [51]. As a part of the participant observation approach, the lead author and her assistant lived with host families in the PNC community for two months. All observations of primate use were recorded. This type of participant observation allows researchers to develop trusted relationships with community members. In addition, it allowed for visits to family homes. Living among the community also made it easier to ask more questions and receive clarification on the uses and perceptions of primates.

### Data analysis

Free listing data were analysed to determine the most salient species, namely those mentioned by more participants in response to each question. A species’ salience was calculated as the percentage of participants who mentioned it in response to a free listing question. Analysis of the responses to open-ended questions in the free listing interviews regarding perceived changes in species abundance and those regarding the consumption of smaller-bodied primate species was made using the coding function in Nvivo 2.0. The codes reflect themes arising from responses, with most responses including 2–3 themes (Table 1). The most frequently mentioned and those which were mentioned together were measured by code counts.

**Table 1:** Main themes arising from responses on reasons for animal scarcity in the PNC community

Code	Items coded	Examples
‘Animals moved away from the area’	Phrases* indicating that animals have moved away from the area	‘The animals don’t come close anymore’, ‘The animals move away’
‘Deforestation’	Phrases regarding loss of forest	‘Because of deforestation’
‘Hunting’	Phrases emphasizing the hunting or consumption of animals by people	‘We finished them’, ‘Because people kill them’, ‘The population grew and consumed more animals’
‘Noise’	Phrases indicating animals are disturbed by noise with three sub-codes used when noise source was mentioned (Logging companies, loud machinery, oil companies)	‘The companies make a lot of noise’, ‘Loggers entered with tractors and made noise’
‘Population increase’	Phrases regarding the increase in number of people in the community	‘The population grows…’, ‘There are more people’, ‘The community grew’

*In this table, ‘phrase’ is defined as a portion of a sentence or keyword that fits under a specific code

## Results

According to triangulated data from semi-structured and free listing interviews, species identification using photo flashcards and our observations, 13 primate species were identified by participants and are found, or used to be found, in and around the PNC community (Table 2).

**Table 2:** Primate species identified by Shipibo informants using photo flashcards and their conservation status

Species	Common name	Spanish name	Shipibo name	IUCN red list status	Population trend
*Alouatta seniculus*	Colombian red howler monkey	Mono koto	*Ro*	LC	Decreasing
*Aotus cf. nigriceps*	Black-headed night monkey	Musmuki	*R(d)iro*	LC	Unknown
*Ateles chamek*	Black-faced black spider monkey	Maquisapa	*Iso*	EN	Decreasing
*Cacajao ucayalii*	Bald uakari	Huapo colorado	*Huxin*	VU	Decreasing
*Cebus unicolor*	White capuchin	Mono blanco	*Juxo*	VU	Decreasing
*Lagothrix lagothricha*	Common woolly monkey	Chorro	*Isokor(d)o*	VU	Decreasing
*Leontocebus weddelli*	Weddell’s saddle-back tamarin*	Pichico	*Shipi*	LC	Unknown
*Pithecia inusta*	Burnished saki	Huapo negro	*Nano*	LC	Decreasing
*Plecturocebus discolor*	White-tailed titi monkey	Tokon	*Roca roca*	LC	Unknown
*Tamarinus mystax*	Moustached tamarin*	Pichico	*Shipi*	LC	Decreasing
*Saimiri boliviensis*	Black-capped squirrel monkey	Mono ardilla	*Wasa*	LC	Decreasing
*Saimiri cassiquiarensis*	Humboldt squirrel monkey	Mono ardilla	*Wasa*	LC	Unknown
*Sapajus apella*	Brown capuchin	Mono negro	*Huiso*	LC	Decreasing

*Tamarin species were inconsistently identified by the informants due to similarity in appearance between species, and identifications in this case were made based on the species observed by the researchers (LC = Least Concern; VU = Vulnerable; EN = Endangered [54])

A total of 43 community members were interviewed, aged 26 to 79 (mean age = 48.8, SD ± 14.64). We interviewed 25 men (mean age = 51.6, SD ± 13.7) and 18 women (mean age = 44.8, SD ± 15.37). Two tamarin species, Weddell’s saddle-back tamarin (*Leontocebus weddelli*) and moustached tamarin (*Tamarinus mystax*), were pooled in the analysis under ‘tamarins’ as participants did not differentiate between them and referred to all tamarins as one type of monkey, *Pichico*. Similarly, black-capped squirrel monkey (*Saimiri boliviensis*) and Humboldt’s squirrel monkey (*Saimiri cassiquiarensis*) were both referred to as *Huasa* and grouped under *‘Saimiri* spp.’ for the same reason. The most frequently mentioned animals seen in the community area and surroundings were tamarins, squirrel monkeys (*Saimiri* spp.) and agoutis (*Dasyprocta fuliginosa*), while species reported as depleted were white-lipped peccary (*Tayassu peccari*), spider monkey (*Ateles chamek*), and woolly monkey (*Lagothrix lagothricha*). The most preferred species for consumption were lowland paca, deer (*Odocoileus virginianus*), and woolly monkey. The most frequently mentioned animals that are avoided for consumption were anteater (Family Myrmecophagidae), jaguar (*Pantera onca*), and sloth (*Bradypus variegatus*). Animals most rarely consumed were white-lipped peccary, tapir, and deer (Table 3).

**Table 3:** Free listing results showing the top 10 mentioned species and the % of mentioning participants

Species	Common name	Salience/% of participants mentioned* (%)
**Question 1—‘Which wild animals do you see around here?’ (n = 40)**		
*Leontocebus weddelli* + *Tamarinus mystax*	Tamarins	72.5
*Saimiri* spp.	Squirrel monkey	50
*Dasyprocta fuliginosa*	Agouti	45
*Dicotyles tajacu*	Collared peccary	40
*Cuniculus paca*	Lowland paca	40
*Plecturocebus discolor*	Titi monkey	35
*Odocoileus virginianus*	White-tailed deer	30
*Tayassu peccari*	White-lipped peccary	22.5
*Sapajus apella*	Brown capuchin	15
*Aotus cf. nigriceps*	Night monkey	12.5
**Question 2—‘Wild animals you used to see in the past but don’t see them at all or not as much these days?’ (n = 42)**		
*Tayassu peccari*	White-lipped peccary	59.5
*Ateles chamek*	Spider monkey	48
*Lagothrix lagothricha*	Woolly monkey	45
*Cacajao ucayalii*	Bald uakari	36
*Tapirus terrestris*	Tapir	36
*Sapajus apella*	Brown capuchin	31
*Odocoileus virginianus*	White-tailed deer	31
*Dicotyles tajacu*	Collared peccary	28.7
*Cebus unicolor*	White capuchin	21.4
*Alouatta seniculus*	Howler monkey	19
**Question 4—‘Which wild animals do you most like to eat?’ (n = 43)**		
*Cuniculus paca*	Lowland paca	70
*Odocoileus virginianus*	White-tailed deer	60
*Lagothrix lagothricha*	Woolly monkey	49
*Sapajus apella*	Brown capuchin	32.5
*Dicotyles tajacu*	Collared peccary	30
*Tayassu peccari*	White-lipped peccary	28
*Ateles chamek*	Spider monkey	25.5
*Alouatta seniculus*	Howler monkey	21
*Penelope jacquacu*	Spix’s guan	21
*Cebus unicolor*	White capuchin	21
**Question 5—‘Which wild animals you never eat?’ (n = 42)**		
N/A	Anteater	43
*Pantera onca*	Jaguar	43
*Bradypus variegatus*	Sloth	26
N/A	Snakes	19
*Cacajao ucayalii*	Bald uakari	12
*Pithecia inusta*	Burnished saki	9.5
*Leopardus pardalis*	Ocelot	9.5
N/A	Porcupine	7
*Tapirus terrestris*	Tapir	7
*Eira barbara*	Tayra	7
**Question 6—‘Which wild animals you don’t eat very often?’ (n = 40)**		
*Tapirus terrestris*	Tapir	20
*Tayassu peccari*	White-lipped peccary	20
*Odocoileus virginianus*	White-tailed deer	15
*Dicotyles tajacu*	Collared peccary	10
N/A	Primates	10
*Chelonoidis denticulata*	Yellow-footed tortoise	10
*Dasypus* spp.	Armadillo	7.5
N/A	All wild animals	7.5
*Pithecia inusta*	Burnished saki	5
*Hydrochoerus hydrochaeris*	Capybara	5

*Percentages total > 100 as listing included multiple species for each participant; N/A = scientific name not applicable or species could not be accurately identified at the genus level

Primates were sometimes reported as a group, as participants insisted they meant all primates and did not want to name specific species. Therefore, primates as a group were included as an item for this question.

**Fig. 2:**
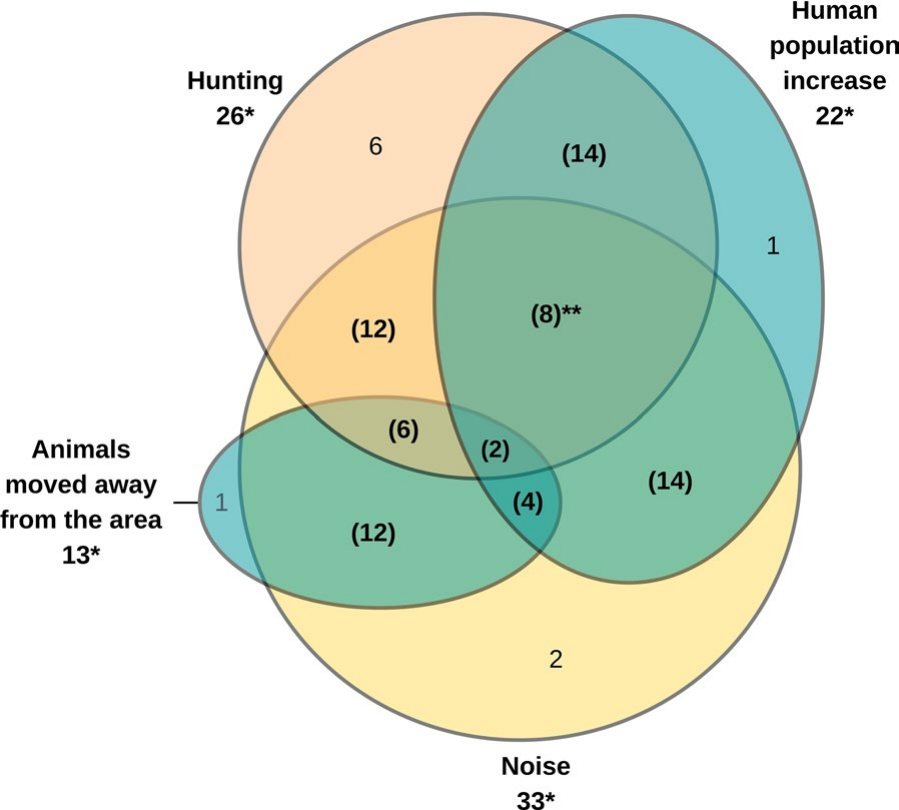
Venn diagram representing reported reasons for animal scarcity and overlapping themes by count of participants who mentioned each theme. Overlapping themes marked by brackets. *The total mentions for each theme. **The size of the overlap does not represent the strength of the relationship

Reported reasons for animal scarcity were coded into five main themes (Table 1). The most frequent reason suggested for animal scarcity was ‘noise’ (77% of 43 participants), followed by ‘hunting’ (60%), ‘human population increase’ (51%) and ‘animals moved away from the area’ (30%). Only two people (4%) mentioned ‘deforestation’. The theme ‘noise’ included three sub-codes for when the nature of the noise was specified or who was making it. Within noise, 42% mentioned ‘loud machinery’ and 39% mentioned ‘logging companies’, while ‘oil company’ was mentioned by one person. Eighty-four per cent of participants mentioned more than one theme in their response, the most common overlaps occurred between ‘human population increase’ and ‘noise’, and between ‘human population increase’ and ‘hunting’ (Fig. 2). Some responses included all three themes, for example—‘*The population grew, they hunt the animals and the logging companies entered and made noise*’ (41, female). Another common overlap was between ‘noise’ and ‘animals moved away from the area’, as all but one participant, stated that animals moved away also mentioned noise disturbance in their response, for example—‘*Because of the people, the loggers enter with their tractors and make a lot of noise, the animals move away*’ (71, male).

### Uses

Food constituted the most important use of primates mentioned by informants and seen in free listing results. Primates were the most frequently mentioned order in the food preference free listing (83% of 43 participants). Of the primates, woolly monkey (*Lagothrix lagothricha*) was the most frequently mentioned (58%) followed by brown capuchin (*Sapajus apella*, 39%), spider monkey (*Ateles chamek*, 31%), white capuchins (*Cebus unicolor*, 25%), howler monkeys (*Alouatta seniculus*, 25%), bald uakari (*Cacajao ucayalii*, 11%), and burnished sakis (*Pithecia inusta*, 2%). Preference was shown for consumption of primate species described as bigger and fatter, and which have more meat. Although not mentioned as preferred for consumption in the free listing and based on survey results, smaller-bodied species, namely tamarins, squirrel monkeys (*Saimiri* spp.), night monkeys (*Aotus nigriceps*), titi monkeys (*Plecturocebus* spp.), were reported to be consumed by 95% of people interviewed (N = 39). Fifty-nine per cent of participants confirmed that these species were consumed less or not at all in the past. Out of the participants who stated they currently consume small-bodied primate species but did not consume them at all or not as much in the past, 52% (n = 12) related it to the lack of larger species. It was repeatedly mentioned that when larger species were common, the smaller species were not targeted, but now community members hunt and consume what they can find, for example—‘*Yes, we eat them. We eat small monkeys because there are no big ones left, in the past there were bigger monkeys*’ (41, Female). Another example—‘*In the past we almost never eat titi and squirrel monkey, same with night monkey, because we hunted woolly and spider monkeys. The tamarins are hunted when someone is very hungry’* (38, Male). Out of the participants who stated they consumed smaller primate species both in the past and present, four mentioned they are hunted when primates are fat, suggesting more seasonal consumption.

**Fig. 3:**
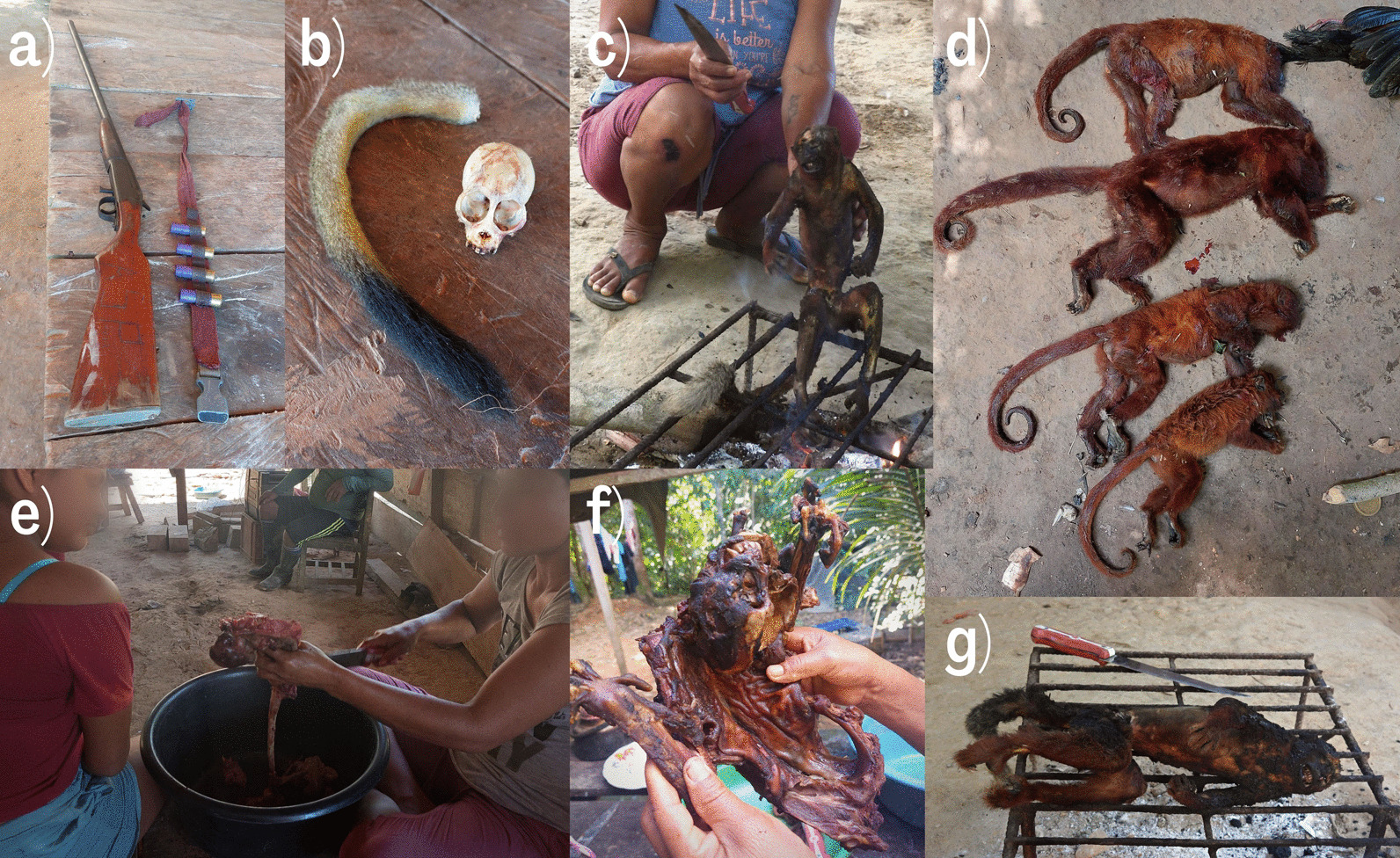
Hunting and preparation of primates and their derivatives. **a** shotgun used for hunting primates; **b** squirrel monkey tail and skull kept in house for medicine and art; **c** titi monkey in the process of fur scraping above open fire; **d** family of howler monkeys hunted upriver and brought by loggers to PNC for consumption; **e** butchering of night monkey to remove its intestines before cooking; **f** primate purchased from commercial hunters; and **g** roasting process

When asked about which species were avoided, 19% of participants (n = 42) mentioned a primate during the free listing; these were mostly bald uakari (12%) and burnished saki (10%), due to a dislike of their smell or taste. Primates were consumed fully regardless of species, with only the intestines and bones discarded. According to participants, primates are hunted using shotguns (Fig. 3a), and traditional hunting with bows and arrows has not taken place in over 20 years. Preparation for consumption includes burning and scraping the fur over an open fire, butchering, and cooking by either boiling, grilling or smoking, a process observed first-hand several times during the study (Fig. 3c, e, f, g). Other forms of consumption mentioned were primate brain with Farina, prepared as a porridge, as well as pudding with tamarin or brown capuchin meat. As well as primates hunted by community members, we also observed consumption of night monkey , squirrel monkey, howler monkey and a brown capuchin purchased from loggers (Fig. 3d, f). During interviews, distaste for primate meat due to its resemblance to humans was often attributed to mestizos (non-indigenous Peruvians), while noting it is an important part of Shipibo culture and the food of their ancestors. One informant (54, male) said that when he and his sons were invited for a meal in the nearby Shipibo village, and served primate meat for breakfast, his sons did not want to eat it because of its human-like hands. In response, the informant told his sons: *‘This is our culture*’. Another informant (59, male) said: ‘*They say that in history the monkey was a human, in the times of the Incas it was like that, they turned people into monkeys and that is why some do not eat because they look like people, mestizos do not eat monkeys, but we do’*.

**Fig. 4:**
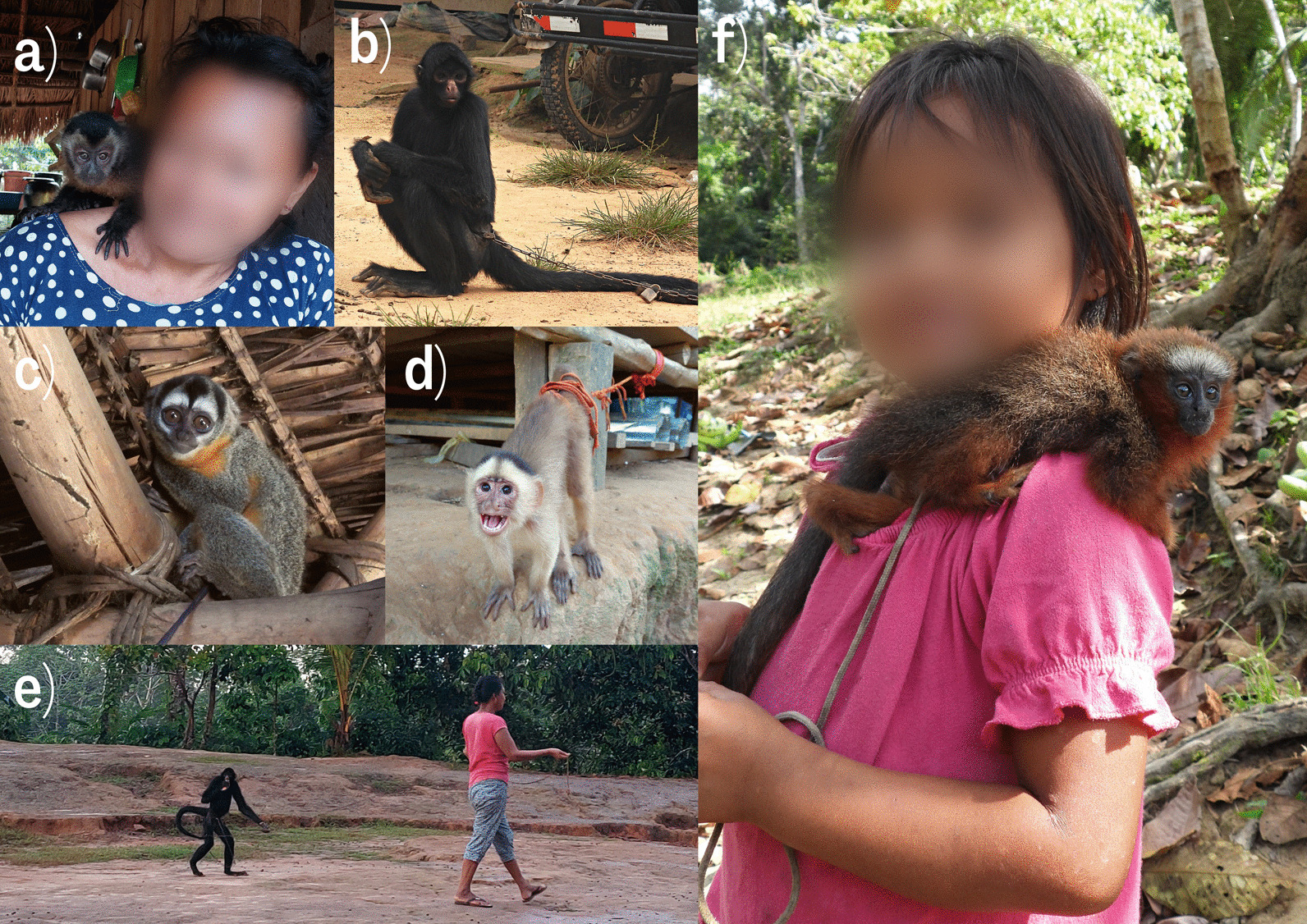
Primates as pets in PNC and neighbouring communities. **a** A woman with her pet brown capuchin, PNC; **b** pet spider monkey restrained by chains, PNC; **c** a pet night monkey a few days after capture, tied to the house ceiling, PNC; **d** white capuchin in the nearby village of Curiaca; **e** an escaped pet spider monkey being taken back to its owners, PNC; and **f** a young girl from San Luis village with her pet titi monkey given to her by loggers

The second most common use of primates by the Shipibo was as pets. Sixty per cent of participants (N = 43) owned or had owned pet primates, with white capuchins being the most common, followed by tamarins and woolly monkeys. Pet primates were either captured live during subsistence hunting or hunted specifically for pet keeping. It was often mentioned that primates are kept as pets by women who like to carry them on their head and hands, as adornment in the house and for their song. The ‘mischievousness’ (*Travieso*) of pet primates was said to ease sadness. White capuchins were repeatedly mentioned as the most mischievous and intelligent primate, and that they often steal or break objects, especially chicken eggs. Bald uakari and brown capuchin were also referred to as mischievous pets, while tamarins, squirrel monkeys, and white-tailed titi monkeys (*Plecturocebus discolor*) were said to be calm and sing beautifully. Night monkeys were also said to make good pets, sleeping during the day, and woolly monkeys were thought to make the most beautiful pets. Participants cited howler monkeys as the ‘strongest’ primates in the wild but indicated that when kept as pets, they become docile and are the noisiest and least resilient primates. Pet primates are also given as presents and sometimes sold because they become too difficult to maintain when they mature, however some participants mentioned that pet primates do not survive more than a few months and do not grow as big as they would in the wild. During the study, five pet primates were observed in the village: a spider monkey, night monkey, brown capuchin, squirrel monkey, and a moustached tamarin (Fig. 4). White-tailed titi monkey and white capuchin pets were also observed as pets in nearby villages (Fig. 4c, e). All pets were reported to be captured locally on surrounding trails, while only the spider monkey was brought by loggers working further upriver, approximately a day by boat. Three pet primates died in the community during the study period. These deaths were from poisoning, infected shotgun wounds, and malnutrition, as explained by the owners. Pets were kept tied by a string, rope, or chain (Fig. 4b, c, e). Informants noted that pet primates are not consumed when they grow older or die, but rather treated as a part of the family and buried. Some informants stated that according to Peruvian law owning a pet primate or selling it is illegal and owners could get arrested, but that this law is never enforced in rural villages.

Primate derivatives were also reported to be used in jewellery and for medicinal purposes. Although use of primate tails for medicine was rarely mentioned during interviews and was observed only once during the study period, tails were reported to be used to reduce pain caused by scorpion, bullet ant (*Paraponera clavata*), and spider bites, by cooking the tip of the tail with a piece of charcoal and inhaling and/or letting the smoke touch the wound. The species of primate used for this purpose was reportedly squirrel monkey, titi monkey or any primate. This use was often stated to be ‘a thing of the past’ and that these days Shipibo primarily use creams and medications from pharmacies. Another medicinal use was cooking the fur of primates to cure ‘sickness’. Primate bones were previously used to make jewellery, mostly bracelets and necklaces from teeth, although this type of jewellery was not seen during the study and informants mentioned they are not worn anymore. Spider monkey arm bones were mentioned to be the most suitable for making tobacco pipes in the past, as they have the most resistant hollows. Skulls were kept in some houses and were observed in tourist markets in the city of Pucallpa, while informants noted that selling skulls is a recent custom, mostly aimed at tourists who ‘buy everything’ (Fig. 3b).

Taboos concerning the consumption of primates were few and said to be rarely practised. It is believed that pregnant women should avoid consuming primates especially in their last trimester as it could make the newborn deformed, mischievous, or a ‘cry baby’, taking the characteristics of the species. It was mentioned that tamarins can be eaten during pregnancy, as the newborns will receive the ‘good face’ of the tamarins. However, we did not observe consumption of tamarins and informants said that they are rarely consumed as they are small and meatless. Male informants who mentioned traditional beliefs about primate consumption during pregnancy consistently said they are rarely practised today and ‘only a belief’, while female participants stated that only a few still practise it. A 64-year-old male informant noted—‘*A pregnant woman would not eat monkey, she didn’t eat any monkey until she gave birth otherwise her baby will be mischievous. This is the belief for us, but they would eat tamarins for their babies to have a good and beautiful face. These are the beliefs of our ancient grandparents almost the first generation*’. Primates were also said to be avoided by shamen on strict diets, however there were no shamen in the community to confirm this.

### Stories and traditional knowledge

Shipibo knowledge of primates reported in this study often described observations from forest encounters and hunting experiences. Two informants mentioned that the bald uakari let their young loose when they feed, providing an opportunity to capture them as pets. Bald uakaris are said to be the ‘fastest’ monkeys. They are seen more often during the wet season, but further upriver, and move in large groups of 20–30 individuals. Informants often made jokes about bald uakari fur, either that the animal is never cold or that below its fur it is pale and skinny. One informant mentioned how he saw a bald uakari fall into the river with its hands outstretched after failing to retrieve fruit from a tree. The monkey got out of the water pale, skinny, and wet, and that due to his feelings of pity and amusement, the informant did not hunt it. Hunting experiences from the times that bows and arrows were used were shared often during interviews. Burnished sakis were said to escape from arrows, while spider monkeys were the biggest and most dangerous monkeys; sometimes catching arrows or pulling them out, and escaping with them, or even throwing them back at the hunters.

According to informants, white capuchins eat smaller monkeys, like squirrel and night monkeys, and therefore, these species are not seen together. Squirrel monkeys are more likely to be seen with other monkeys, as they go in big groups and protect each other, while night monkeys, which live in small family groups, are afraid of the white capuchins. Black-capped squirrel monkeys are seen with the brown capuchins and live in bigger groups than the Humboldt squirrel monkey. Some primates were said to never go together, for example, woolly monkeys will never mix with howler monkeys as they are similarly coloured and because the woolly monkey is much faster and ‘alive’ while the howler monkey is slower and lazier. Howler monkeys were also said to howl in the mornings and when rain is coming.

Informants tended to assign human characteristics to primates. For example, spider and howler monkeys were said to frequently fight because they ‘cheated’ each other in their past. One story tells how the howler monkey tricked the spider monkey, pretending to have only four fingers, convincing the spider monkey to be like him and cut one to match. After the spider monkey cut his fifth fingers off, the howler revealed his trickery. As an act of revenge, the spider monkey told the howler monkey ‘*My beautiful song is heard from far away, I can make you sing like me*’, telling him to place the huingo fruit (*Crescentia cujete*) in his throat. The huingo got stuck, making his song a loud low howl. In another version, the spider monkey switched his voice with the howler monkey, tricking him and saying he will give him his ‘flute’ after which the howler monkey remained with his ‘thick’ and ‘ugly’ voice.

### Myths and traditional beliefs

According to the Shipibo in PNC, all primates were once humans. Numerous participants discussed their traditional beliefs on the origins of primates and humans while acknowledging they contradict ‘Western’ science and Christian doctrine. A 70-year-old community member related that ‘*Scientists say that we come from the monkeys, but the Shipibo are very different. The history of the ancestors says that we are not from monkeys, rather, the monkeys come from us, humans transformed into monkeys*’. One of the consistent myths about primates we heard links the creation of primates to how the Shipibo received their name: there were two Inca people in the Shipibo land—the good Inca from which the Shipibo descended, and the bad Inca, who had the power to transform humans into animals. The two Inca fought all the time as the bad Inca never wanted to share his food. In one version of the story, the bad Inca got angry at the local people for drinking too much of their traditional fermented drink, Masato, which left white foam around their mouths. Thus, the bad Inca transformed them into tamarins, known as *shipi* in Shipibo, followed by *bo* to indicate the plural. Another version tells that the Shipibo who drank Masato often did not wipe their mouths and looked like tamarins and began calling each other, or were called by others, *Shipi*. In another version, Shipibo who didn’t have enough food used to eat cooked huito (*Genipa americana*), which stained their mouths black, and the bad Inca then transformed the people into monkeys and that is why squirrel monkeys, tamarins, and howler monkeys have black mouths. A similar myth regarding squirrel monkeys says that children used to play with and eat the fruits of the bad Inca who shouted in anger, ‘*You are mischievous and will remain Huasa*!’ and transformed them into squirrel monkeys. According to another myth, primates, which were previously human, taught the Shipibo to have sex as the Shipibo people did not know how to multiply and grow their population. In this myth, the Shipibo did not have sexual relations even though women and men lived together. One day, a monkey went to a man and asked, ‘*What are you doing with your woman?*’ He replied, ‘*looking at her*’, then the monkey had intercourse with the woman and told the man ‘*This is how you do it, now you try it*’. Since then, the Shipibo started to have sex. After telling the story, the informant added—‘*The monkey taught the man because he himself was formerly a man. This is why they say that we are monkeys, but the religious theory says no, we are creation of god. After that the Shipibo started to have children, because before they did not know how to multiply’*.

The transformation of primates back into humans was also mentioned often during interviews. According to one belief, primates transform into humans to attend parties, looking and speaking exactly like humans but refusing to drink Masato as their identity might be revealed. Other beliefs state that primates, transformed into humans, appeared in village houses, speaking with the women and kidnapping them and later transforming back into monkeys. The women always tried to run away from their kidnappers because of their hairy bodies when they were monkeys. Furthering the idea of transformation and, as primates are a source of food, EA inquired weather consuming primates that were previously humans is considered as a form of cannibalism. However, all informants denied any link to cannibalism and said that since primates were transformed, they are no longer human. Some informants also mentioned that the Shipibo have never performed cannibalism, unlike their former enemies the Cacataibo, who would eat their enemies.

Another belief, reported by four informants, was about a large human sized monkey that lived far away in the mountains and was rarely encountered. This monkey was described as a big spider monkey (*Iso Ewa/Mashi iso*) with a 5 m tail that it used for kidnapping people. This big primate was said to never harm the people it took, treating them well as a husband or wife, and feeding them with fruits. However, the Shipibo were scared of encountering it, as they knew they would never return to their homes. The four informants who related this story mentioned they had never seen the big monkey but learned about it from their grandparents who had seen it and warned them about it. When informants tried to describe the big monkey, they used terms such as ‘gorilla’ and ‘chimpanzee’ as examples, while acknowledging they exist only in Africa.

## Discussion

Indigenous people inhabit lands that protect most of the world’s remaining biodiversity and over 70% of primate habitats [8]. The importance of their traditions and knowledge systems is recognized as key to sustainable land management and the conservation of the world’s remaining biodiversity [8, 22–24, 26]. However, outside pressures, integration into market economies and population growth, are raising doubts regarding the sustainability of traditional practices and their role in conservation [55]. Indigenous people and their territories play an essential role in primate conservation through ecosystem preservation [8, 26]. In this paper, we documented the important role primates hold in Shipibo culture and subsistence through qualitative data gathered via free listing, semi-structured interviews and participant observation. We discuss our findings, limitations, and implications in further detail under the topics covered and end with a positionality statement and conclusions.

### Depletion and animal scarcity

Free listing interviews allowed us to see which animals are present and absent from the local environment based on local knowledge, without relying on biological survey data. White-lipped peccary, spider monkey, woolly monkey, and bald uakari were perceived as the most depleted species and were reported as being previously abundant and even observed within the community settlement 10–20 years ago, but rarely seen today. Based on community member reports and our own observations, spider and woolly monkeys are likely locally extinct as seen around other indigenous communities [34, 56]. Participants under 30 years of age did not mention spider or woolly monkeys in the depletion question. This may suggest that these species have not been seen in the area for an extended period, however there are no prior studies conducted in the communities of the upper Ucayali for comparison.

One aim of our study was to describe and highlight local indigenous views and explanations for animal scarcity, recognizing their knowledge and understanding of their own territory. Most participants reported more than one reason, and the results indicate that Shipibo community members in this study mainly believed it is a combination of noise disturbance, hunting practices and population growth that has led to depletion. Noise was the most common theme and often linked to extracting companies and machinery. Noise and overall human presence can affect primate behaviour, even more than spatial disturbances [57]. However, further investigations are needed to determine in more detail what types of human presence and noise in particular could cause significant shifts in habitat ranges to a point of abandonment, as reported by participants, and which species are affected. The views on hunted animal and fish depletion among Shipibo were reflected in the myth stories documented by Cabrera [58] in the 1980s. One myth about the creation of hunting tools ends on a pessimistic note that ‘merciless’ mestizo hunters are exterminating the river turtles and leaving deserted beaches along the Ucayali River ([58], pp. 78–81). While comments about population growth in the present study also referred to mestizos and non-indigenous people, only two participants related the animal scarcity to mestizos. Logging companies, which were often mentioned by the participants as a source of noise and cause of depletion, come with the presence of non-indigenous people in the forest and their presence should be explored in future studies. The Shipibo historically were dispersed along the Ucayali region but with a growing population and number of communities, have become increasingly sedentary [46]. Further to the community’s mentions of population growth and animal scarcity, a notion about past migratory practices among the Shipibo was also made. One of the village elders said that historically the Shipibo migrated from place to place, made temporary homes where fish and animals were abundant and, when they noticed scarcity, they left looking for another site to live. Increasing sedentarism and reliance on agriculture by indigenous people in tropical forests, affects hunting sustainability and leads to local depletion as seen in contemporary Shipibo and other Amazonian groups [59, 60]. Throughout the interviews, community members expressed concern about the depletion of wildlife. Some participants discussed empty rivers and that they rarely catch fish with a rod but must use nets, or that some local children never had a chance to eat *paiche* (*Arapaima gigas*), the largest fish in the Amazon, that is now gone from the rivers and that favoured primate species are not consumed as much anymore as they are harder to find and that simply ‘*there aren’t any/ya no hay*’. Participants demonstrated interest and desire in Aquaculture, as a potential solution to depleting food resources. Sustainable aquaculture was also prioritized by the PNC general assembly as seen and recommended in other parts of the Peruvian Amazon and could help reduce pressure on local fish and hunted species and produce additional income for local people [61, 62].

The impact of shotgun hunting on species depletion, especially primates, goes beyond the impacts of population growth and settlement spread [61]. The transition from traditional hunting methods was previously documented among Shipibo communities in Ucayali, with Morin [46] noting the shift from blowguns to bows and arrows more than a decade ago, and Behrens [47] the shift to shotguns over 40 years ago, changes which were also linked to changing food patterns. While bow and arrow and gun hunting differ in their efficiency across species and animal sizes, the use of shotguns has allowed more successful hunting of larger species, such as tapir, among Shipibo [47]. Changes in hunting technology along with the loosening of cultural taboos can lead to a broader diet. Taboos appear flexible and reflect changes between generations and communities; however, the role of taboos in conservation and their impact on wildlife may be difficult to evaluate without historical data on practices over time [63].

The frequency of wild meat consumption varied between families and based on our observations, may depend on household, socio-economic status, preference and season, but could not be successfully calculated in our study. Some families without a hunter in their household purchased wild meat from people who work in logging concessions or illegal coca plantations further upriver where animals were said to be more abundant. Purchasing of wild meat was observed during our study and reported by various community members, providing evidence of illegal commercial hunting happening in the area. As commercial hunting has increased in the Amazon [38, 40], understanding its patterns and drivers in the area may help direct where conservation efforts should focus. Further investigation using predictive models of primate depletion to assess the sustainability of subsistence hunting among Shipibo communities could help measure its impact on primate populations and other hunted species [39, 56]. To understand and prevent further depletion, a similar approach that values indigenous knowledge and experience of their own environment and culture would be enlightening. Such approaches can guide conservation through community-based participatory research that empowers and involves local stakeholders in a meaningful and equitable process, actions and active decision making based on culture and needs [64]. This inclusive approach, where the opinions and ideas of indigenous people are ‘heard’, is also crucial for their engagement with conservation efforts [19, 65].

### Food preferences

Although the Shipibo diet consists mostly of fish, rice, and crops such as plantain, manioc, maize, corn, and beans, hunting still plays a significant role. The most preferred animals for consumption were pacas, deer, primates, and peccaries, as also shown in previous studies of Shipibo diets and hunting preferences [46, 66]. However, specific primate species were not mentioned as preferred foods in previous studies among Shipibo. Primates were the most preferred order reported in our study, with woolly monkeys and brown capuchins the third and fourth most preferred species. Woolly monkeys and brown capuchins have been reported as the preferred species by other indigenous groups in the Amazon, such as the Tikuna [10], Secoya [43], and the Kayapo [67]. Overall, among indigenous Amazonians, primates are preferred targets, especially atelines due to their size and tasteful meat [68]. Primates in general were also reported by community members to be consumed rarely because they are harder to find. As noted by Peres [34], indigenous Amazonians consistently prefer larger mammals and bird species, a finding mirrored in our study. However, we found that lowland paca was the most preferred species for consumption, even though it is not one of the largest species and has not been mentioned as a primary species in previous reports on Shipibo hunting preferences and diets [46, 47, 66]. As multiple factors affect the mentioning of items in free lists, such as memory, familiarity, and prominence [50, 69], the indicated preference for lowland paca could have been a result of its availability rather than consumption preference. Some of the most frequently mentioned species that our participants stated they avoid, such as sloth, jaguar, and snakes, were also classified as animals avoided for consumption by the Shipibo in a previous study [47]. After listing the avoided species, participants often added that their ancestors and grandparents never used to eat these animals, that the Shipibo are not eating them or simply avoided due to bad taste. Behrns [47] documented Shipibo food categories where avoided foods were classified under ‘*Rambi jahuëki*’ referring to anything in the environment that is ‘ugly’, ‘not useful’ or ‘can hurt you’, while in our study no such category or any food category was named or mentioned. Other avoided species in Behrens’ study included eels, capybara, and kinkajous which only one or two participants stated were avoided in our study. This suggests, as Behrens [47] stated, that diets become more inclusive as the Shipibo accept previously prohibited or avoided species into their diet.

The preferred primate species were the most targeted and likely driven to local extinction, except for the brown capuchin (Anca, unpublished data). While spider and woolly monkeys were said to be the most delicious, almost 50% of participants named them as depleted species. With the disappearance of large-bodied primates around PNC, the availability of primate species for consumption may have influenced the free listings for preference, as answers could have been biased by memory and availability [69]. Depletion may also influence reports from younger participants who grew up without the presence of larger species, who did not consume them as much as older participants, to be established as a preferred or commonly consumed species on either a cultural or individual preference level. The small sample size (N = 43) and strict Shipibo origin of all participants may not be representative of the entire community and its mestizo immigrants. A more inclusive sample may identify changing dietary patterns and the impact of the community as a whole on local wildlife.

### Consumption of smaller species

During the study, white-tailed titi monkeys, Weddell’s and moustached tamarins, were often observed around the PNC community. These species were not mentioned at all as a preferred food, but the consumption of white-tailed titi monkeys and other smaller species, such as night monkeys and black-capped squirrel monkeys, was observed. Overall, these smaller-bodied species are consumed but were not regularly consumed in the past, when larger species were available. As ‘in the past’ is a broad concept term we used in order to pick up on a shift in consumption, it is not possible to trace back to when the shift in consumption started. Shipibo informants emphasized that hunting smaller primate species usually is not worth the effort as it cannot feed a family, but today they hunt what they can find. These findings are in line with other findings on increasing consumption of smaller bodied, less preferred species, which were ignored by hunters when larger species were available [34, 36–39]. Participants that claimed they did consume smaller species in the past emphasized the times of the year in which fruits are available and animals are fatter and, therefore, have more meat. In this case, seasonal consumption may help these primate populations to recover, while larger species are targeted year-round. Regardless of seasonal hunting, smaller species are often seen at higher densities near indigenous communities [35, 36] and may be less prone to hunting pressure than larger-bodied species, but understanding trends in their populations and the impact of humans on their habitats and life cycles will help prevent further local extinctions of primates from hunted areas.

### Ethnoprimatology

Our study is the first to provide an overview on the uses of primates among the Shipibo and their portrayal in mythology. Through this ethnoprimatological approach, we highlight the role of primates in Shipibo culture and folklore as well as the interconnections between mythology and traditional beliefs to contemporary views on primates.

The uses of primates and their derivatives for subsistence, as pets and in jewellery, are often seen in Amazonian groups [29, 30]. Primates are preferred pets among Shipibo and seen as an important adornment or companion, especially for women and children. The mentions of primate pet keeping being illegal do not seem to bother pet owners, as it is not enforced very often in remote communities such as PNC. The use of primate derivatives is fading and rarely practised in PNC; however, traces of these customs exist through reports and first-hand observations. Medicinal uses, such as the preparation of primate tails by boiling as reported to EA in PNC, was also documented in the Maijuna for treating digestive problems [12]. Other Amazonian groups have used the tails of primates as dusters [11, 12]. The tails of primate species are also used as adornment for the head or arms among the Yanomami and the Awajun/Aguaruna [32, 70]. While jewellery or wristbands made of primate teeth were not observed during our study, this use was reported by multiple community members and was also documented in the 60s by Morin [46]. Restrictions on primate consumption documented 40 years ago were practised after birth, by both parents, and specifically included avoidance of white capuchins [47]. The only taboo on the consumption of primates reported in our study was by pregnant women but did not mention a specific species. Similar taboos regarding primates applied to fathers of newborns and pregnant women have been documented among the Kayapo, Yanomami, Siriono, and Tapirape [63]. Although Behrens [47] notes that monkeys with young were traditionally prohibited among Shipibo, they were regularly hunted and consumed during his study. It seems that traditional beliefs and practices, such as the restrictions on hunting primates with young or on consumption by pregnant women and their husbands, could help reduce increasing pressure on primate species but are rarely practised in present-day Shipibo culture.

Numerous Amazonian groups share the belief that primates originated from humans who were transformed by a powerful creator [9, 12, 18, 71]. These transformations are often a result of punishment for certain behaviours which are seen as bad by the creator. These repeating concepts of punishment in creation stories may reflect cultural values. For example, the drinking of Masato is a part of Shipibo identity and a custom that persists until this day; however, some were said to be ‘punished’ by the bad Inca for doing so and transformed into primates. Although the transformation into a primate is considered a punishment, there is no evidence that primates are seen negatively. Rather, the specific characteristics or actions that led to their transformations, such as eating fruits on trees, having white or black faces, or being lazy are explanations of the origins of animals and their characteristics in such a biodiverse environment.

The ‘looking like primates’ concept is mentioned in various sources and literature about Shipibo mythology. In Cabrera [58], one myth details the first settlement of the Shipibo which was guided by the sun *Bari* and the *Alto Mueraya* who showed them where to settle before disappearing into the forest. The *Alto Mueraya*, the great shaman, has the power to travel through the world of water, fire, and wind, the abode of the sacred ancestors for guidance to cure people from illness and bad spirits. The settlement fell victim to the bad spirits of the forest that caused illness, sadness, and death. In those times of despair, the *Alto Mueraya* incarnated in the body of a tamarin, *Shipi*, and told the people that his travels in the spirit world revealed that the bad spirits had taken over all natural beings except the tamarins, which remained free. Therefore, if the tribe transformed to look like tamarins, they would be saved. The men then painted their bodies and learned to whistle like the tamarins and the women cut their hair in the shape of tamarin heads. According to this myth, the effort to look like tamarins brought the Shipibo their name ([58], pp. 14–19). In one of the earlier ethnographic studies, the past habit of painting faces black with ‘huito’ paint made them look like the monkey they call *shipi*, tamarin [46]. However, in our study the white spume caused by Masato drinking and the similarity to moustached tamarins was mentioned more often. Myths are never exact reprints, they are ‘alive’ and tend to renew and transform based on time and place, the teller, and audience, while keeping their core meaning [72]. While some details in the Shipibo myths may have changed over time and vary between local populations and samples, the main concepts of the myths remain the same, predominantly the continuity between humans and primates.

Cabrera [58] also wrote about the birth of primates and the transformation of humans into primates. In one myth, that also occurred in the time of the bad Inca, who was the governor and lord of fire and domestic plants and never wanted to share his food. The story details how the children could not bear their hunger and climbed the Inca’s fruit trees. When they were caught, the Inca transformed them into various monkey species: white-tailed titi monkeys, howler monkeys, white capuchins, brown capuchins, woolly monkeys and squirrel monkeys. This myth continues, relating the vengeance of the children’s parents, which resulted in the birth of birds ([58], pp. 117–122). Similarly, in a myth documented by Bertrand-Rousseau [73], the children caught in the tree were transformed into brown capuchins and squirrel monkeys, which were said to treat each other as cousins and live together in a group, and the rest of the children were transformed into the ‘solitary’ white capuchins [73]. Mixed groups of brown capuchins and squirrel monkeys were reported by informants in our study. Community members reported white capuchins as predatory, saying their presence would scare off other primates. In addition, the similarity of primates to humans, which was often acknowledged by community members, is easy to understand when the community believes that human children that were more similar in size to primates were once transformed into them.

According to mythology documented by Morin [46], the Shipibo learned about sexuality, birth, and the use of flutes from the white capuchin. One myth from the literature links white capuchins with sexuality and the birth of jealousy. This myth is called ‘The White Monkey Who Showed us Jealousy’ in Landlot [74] and refers to the past in which Shipibo men shared wives and lived without jealousy and how it all changed when the white capuchin thought the Shipibo about betrayal, jealousy and to react violently to it. It is also mentioned that white capuchins are the most mischievous primate, playing with their private parts and lifting women’s skirts. White capuchins were often referred to as the most mischievous primate in PNC, however, the myth we documented about the monkey teaching the Shipibo to have sex did not specifically mention white capuchins. While the white capuchin’s mischievousness may be linked to sexuality among Shipibo, over time details are lost through myth retelling and tranformation, making it harder to decipher the origin of Shipibo beliefs.

Myths are a ‘body of explanation’ on human existence, the origin of a group and the dependency on the natural world, which are all essential to understanding cultural identity [72, 75]. Myths are often ‘solving’ and interpreting life paradoxes experienced by a group, which justifies traditional beliefs and creates guiding rules for a society [53, 72]. One of those paradoxes could be the physical similarity between humans and primates, related in Shipibo myths by the human origin of primates. The transformation as an element of punishment may provide a cautionary component to these myths, for behaviours and activities humans should not engage in, such as stealing food, climbing trees to eat fruits, drinking Masato, etc.

The belief in the existence of a large monkey that is closely related to spider monkeys and lives in the mountain foothills was reported in this study and is also held by the Matsigenka [18]. For the Matsigenka, this creature was perceived more as a dangerous demon that can kill humans, while the *Mashi Iso* of the Shipibo took humans and never harmed them but rather treated them as husbands or wives. Another similarity with Matsigenka folklore on primates is the joint appearance of spider and howler monkeys. In this story too, their relationship was characterized by deceit and revenge that resulted in the howler monkey’s howl [18]. In both stories, of the Shipibo and the Matsigenka, the primates are anthropomorphized, and explanation is given to the howler monkeys’ song. These similar stories could be explained by the geographic proximity between groups in Eastern Perú or in the ‘big monkey’ case, the mere existence of such a larger primate.

The changes in traditional practices go beyond hunting methods to other customs and materials used in PNC. Apart from reports of medicinal use of monkey tail being practised very rarely, various examples of other cultural changes were also observed. Traditional Shipibo houses are made entirely of natural materials, wood and palm leaves, for the roof, which are slowly being replaced by corrugated metal sheeting. The Shipibo clay ceramics, used traditionally for drinking Masato and other foods and drinks, were rarely seen or used during the study period, and are widely replaced by plastic dishes. Fabrics for the making of traditional Shipibo textiles and clothing are often bought from the city instead of woven from home grown cotton. One informant said, ‘*We used to sweat and work hard for our clothing, houses, and ceramics, but today everything is easy and cheap, much faster to buy than to make*’. During our study, community members voiced concerns about the loss of cultural identity among young Shipibo and their lack of interest in traditional customs, as also observed by Esponiza [76]. Some community members mentioned that their children want to look and dress like mestizos. In regard to hunting, none of the informants owned traditional bows and arrows, but only shotguns. Traditional harpoons for caiman hunting were still present in the PNC community. Fishing is most commonly done using nets due to the depletion of fish in the river and the difficulty of rod fishing. The introduction, and extensive use, of plastics and metal roofing is leading to pollution in the river, settlements, and inside the forest itself and around the PNC community, as observed first-hand during the study period. The changes in cultural identity, traditions, and customs affect not only the wellbeing and empowerment of the people, but has implications for the health of their ecosystem.

Although our sample size was not large enough to reach saturation in semi-structured interviews [77] on primates and their cultural significance, we were referred to a small number of informants who still had this knowledge. The few remaining elders are still familiar with myths and cultural beliefs or able to share knowledge on primates. As younger generations are said to ‘*not want to be a Shipibo anymore’*, increasingly engaging with ‘Western’ culture and less with traditional knowledge, storytelling practices as transmitted from one generation to the next are being lost. Elders noted that as young people become more like mestizos, they do not want to eat primates because of their similarity to humans, which could help conserve primate populations. At the same time, the cultural importance of primates decreases with the loss of cultural identity and depletion of local primate species, and community members may be less inclined to preserve primates. Globalization often demands the formation of a multicultural identity as opposed to a single cultural tradition; Jensen [78] notes the ‘gains and losses’ accompany this process, as some aspects of traditional cultures are ‘left behind’ to allow better adaptation to current multicultural realities [78]. Recent ethnography by Dyck [79] emphasizes the adaptability and resilience of Shipibo culture over time and on multiple levels, from mediums of traditional art to agricultural methods, economic and environmental circumstances, as well as interface with other cultures [79]. In addition, indigenous cultures and knowledge systems are dynamic, constantly adapting and responding to the changing world through adaptive resource management [23]. The current realities of the upper Ucayali highlight the need for securing land rights, self-governance, and agency, which are critical for the resilience of culture and traditional knowledge systems among Shipibo and other indigenous groups that are also key to conservation success [23, 79].

Future research could endeavour to record Shipibo myths, as they are unwritten yet important parts of indigenous history and culture and reflecting its uniqueness in world views and interactions with other peoples and the natural world. With increasing development around Shipibo communities, cultural changes, and adaptations, these myths and their subsequent knowledge are at risk of being lost.

### Positionality

The role of positionality and its importance for conservation outcomes, especially when working with indigenous and local communities, is increasingly recognized [65]. We wish to acknowledge our positionality and that of EA, the lead researcher, during her fieldwork and the biases that may have affected the way this study was interpreted. The lead author is a Western woman, vegan, environmental activist, conservationist, and primatologist; as such, animal welfare ideologies regarding the humane slaughter of animals and views against wild pet keeping can conflict with preserving cultural heritage and biodiversity through human wildlife coexistence and cultural preservation. Primates in distress, hunted and prepared for consumption, as well as being violently restrained, could have triggered reactions and emotions that may have affected the research from data collection to interpretation. Despite following a vegan diet, as a part of EA’s intention to immerse herself in Shipibo culture, she consumed the food offered, including wild meat, although her obvious interest in primates may have led to primate meat never being offered to either EA or her assistant. The same effect could have determined the quality and extent of information shared regarding primate consumption. The presence of a research assistant, a Peruvian national, could also have affected the interactions and level of engagement with community members. We acknowledge the undeniable influence of power dynamics on this study, resulting from both previous history with outsiders and the presence of EA, a postgraduate student with means to reach the Amazon to research and document Shipibo culture and their interactions with primates. These imbalances can inherently influence the willingness, discomfort or hesitation to share cultural knowledge and personal views and ultimately, shape the nature of the relationships created with community members. Spanish is not the mother tongue of the participants, who speak Shipibo among themselves, while it is also not the mother tongue of EA. This language barrier probably affected the input from participant observation, the interpretation of information shared during interviews and communication as a whole. To achieve our goals and expand the knowledge on the roles of primates in Shipibo culture that can inform inclusive conservation approaches, we had to juggle the different roles of the first author, and with every step determine which role would lead to the best outcomes for our data. There is always the risk of bias due to personal and cultural preconceptions but reflections on positionality can help us and the readers of this work better understand our perspectives and what influenced them [65, 80].

## Conclusions

Human–primate interactions in the upper Ucayali River occur in intertwined sociocultural levels, as primates are an integral part of social practices, oral traditions and seen as a component of cultural identity. Primates are preferred pets and food source among the Shipibo, and their characters and history are portrayed in mythology and folklore. Previous records in parallel with our results demonstrate that with increasing exposure to ‘Western’ society and the process of acculturation, Shipibo traditional knowledge and the cultural significance of primates erodes as older generations pass away. Growing pressures of globalization on Shipibo, expanding populations, and increasing integration into market economies can lead to increasing depletion of local primate populations, with larger-bodied species quickly becoming locally extinct. Hunting using non-traditional methods and industrialized extractive exploitation of natural resources in the area impacts both the natural environment and the people living there. Insights into the role of primates in Shipibo culture should continue to be gathered and can be used to assist participatory community-based conservation efforts as they provide cultural narratives which may help bridge gaps between the conservation of primates and Shipibo culture, community and identity. To our knowledge, this is the first study to focus on the significance of primates in Shipibo culture and provides a valuable reference for future conservation initiatives aimed at protecting the rich primate diversity and its cultural value for the Shipibo, the third largest indigenous group in the Peruvian Amazon.

## Data Availability

The datasets used and analysed during the current study are anonymized and available from the corresponding author on reasonable request.

## Abbreviations

IUCN: International Union for Conservation of Nature
NPC: Neotropical Primate Conservation
PNC: Comunidad Nativa Pueblo Nuevo del Caco
SD: Standard Deviation
TEK: Traditional Ecological Knowledge
UK: United Kingdom
